# The Role of the Private Sector in Advancing Antimicrobial Stewardship: Recommendations from the Global Chief Medical Officers' Network

**DOI:** 10.1089/pop.2020.0027

**Published:** 2021-04-09

**Authors:** Elizabeth D. Hermsen, Richard Jenkins, Ivo Vlaev, Steve Iley, Thirumalai Rajgopal, Jonathan Mark Sackier, Pietie Loubser, Nico Pronk, Elizabeth Wilkinson, Yat Chow, Cathryn Gunther

**Affiliations:** ^1^ Global Population Health, Merck & Co., Inc., Kenilworth, New Jersey, USA.; ^2^ Medical Services, Emirates Airline, United Arab Emirates.; ^3^ Warwick Business School, Coventry, United Kingdom.; ^4^ Jaguar Land Rover, Warwick, United Kingdom.; ^5^ Global Medical and Occupational Health, Unilever, Mumbai, India.; ^6^ Helius Medical Technologies, Newton, Pennsylvania, USA.; ^7^ Mediclinic Middle East, Dubai, United Arab Emirates.; ^8^ HealthPartners, Minneapolis, Minnesota, USA.; ^9^ British Airways PLC, London, United Kingdom.; ^10^ Quality HealthCare Medical Services, Hong Kong, China.

**Keywords:** population health, health care, antimicrobial resistance, antimicrobial stewardship

## Abstract

Antimicrobial resistance (AMR) occurs when microorganisms develop the ability to defeat the drugs designed to kill them. If allowed to increase at the current rate, AMR could kill an estimated 10 million people per year and cost society approximately 100–200 trillion USD globally by 2050. The slow development of novel antimicrobials further exacerbates the problem. Most human antibiotic use occurs in homes and workplaces, where antibiotic-resistant infections may contribute to diminished performance and loss of work productivity. Employers in the private sector have the ability to reach large populations of employees and their families, raise awareness about AMR, and promote antimicrobial stewardship (AMS) among their workforce. The authors describe 4 steps a company can take to help advance AMS: (1) sign the AMR Pledge, (2) perform a gap analysis, (3) implement and/or modify standard practices, and (4) measure and report outcomes. Real-world examples are provided, including barriers faced, in order to successfully implement initiatives to promote better AMS. Behavioral methods to influence change in the workplace are also presented. Both large and small companies can make a difference to support responsible use of antibiotics and improve the health and well-being of their employees.

## Introduction: Why Employers Should Care About Antimicrobial Resistance

Antimicrobial resistance (AMR) is considered by the World Health Organization (WHO) to be a threat to global health security, killing an estimated 700,000 people across the world every year.^1^ AMR occurs when microorganisms change so that antimicrobials, which include antibiotics (antibacterials), antifungals, antivirals, and antiparasitics, are ineffective at curing the infections caused by the pathogens. In the future, simple surgeries, such as joint replacements, may become increasingly risky because of the spread of AMR.^2^ If allowed to grow unchecked, AMR will kill an estimated 10 million people per year globally—more than cancer and diabetes combined—and cost society approximately 100–200 trillion USD by 2050.^1,2^ Antimicrobial use and insufficient hygiene are key drivers for the development of AMR, with global trade and travel fueling the problem.

Compounding the problem, the development of antibiotics (in particular the discovery of new drug classes) has slowed to an unprecedented rate and has been a growing concern for some time, drawing global attention from various stakeholders.^3,4^ Although antibiotic approvals have increased recently, future research and development of novel antimicrobials will be minimal without additional economic incentives. Therefore, responsible use of antimicrobials, or antimicrobial stewardship (AMS), is imperative. AMS refers to coordinated strategies to improve the use of antimicrobials, with the goal of enhancing patient outcomes and minimizing unintended consequences of antimicrobial use, such as side effects and AMR.

Eighty to ninety percent of antibiotic use in humans occurs in the outpatient setting, including homes and workplaces,^5^ where approximately 30% of antibiotic prescriptions are considered inappropriate or unnecessary.^6^ Moreover, in many countries, individuals can purchase antibiotics without a prescription.^7,8^ These medications may be counterfeit and/or contain impurities that may lead to serious side effects. Indeed, antibiotics and antimalarials are two of the most common drug classes reported to the WHO as falsified or substandard.^9^ As global travel for business and leisure becomes more common, the potential to further exacerbate the problem of AMR exists.^10^ People with antimicrobial-resistant infections or serious side effects from antimicrobials may face long hospital stays and suffer from a loss of productivity and income,^10^ causing individuals, families, the workplace, and communities to suffer.

AMR is a complex topic with a high prevalence of misperceptions and misunderstandings.^11^ Increased awareness and understanding of responsible antibiotic use, infection prevention, and AMR allows individuals to be good stewards of antibiotics, make informed decisions for their personal health, and remain in the workplace by reducing absence due to sickness. The private sector can both help with and benefit from AMS.

## Role of the Private Sector in Antimicrobial Stewardship

As AMR continues to plague the global health community, multi-sectoral efforts are essential.^11^ Several initiatives have begun globally, at the national level, and in local communities,^12–17^ but more is needed.^18^ The private sector comprises 90% of the jobs worldwide,^19^ with a tremendous capacity to reach many people. Employers in the private sector can play an important role in raising awareness about AMR and promoting AMS among their employees, and in some cases, the populations they serve. All companies can educate employees about infection prevention and responsible use of antibiotics, facilitate employee immunizations, encourage hand hygiene, and support employees to stay home when they are sick, thus decreasing the spread of infection and the need for subsequent antibiotics.

AMS is relevant to both the personal health and well-being of employees and the worldwide community. Antimicrobials are the only class of medication for which one person's use can affect the effectiveness of the medication in other people. With most antibiotic use occurring in the community setting,^5^ and workplaces serving as proxy communities, efforts to increase awareness and improve infection prevention among employees are critical.

This paper presents 4 steps companies can take to help advance AMS, including real-world examples from a variety of companies: British Airways, Bupa (examples of Quality HealthCare), Emirates Airline, HealthPartners, Helius Medical Technologies, Jaguar Land Rover, Mediclinic Middle East, Merck & Co., Inc., Kenilworth, NJ, USA (known as MSD outside of the US and Canada), and Unilever (Table 1).

**Table 1.** Company Profiles

## Step 1: Sign the AMR Pledge

**FIG. 1.**
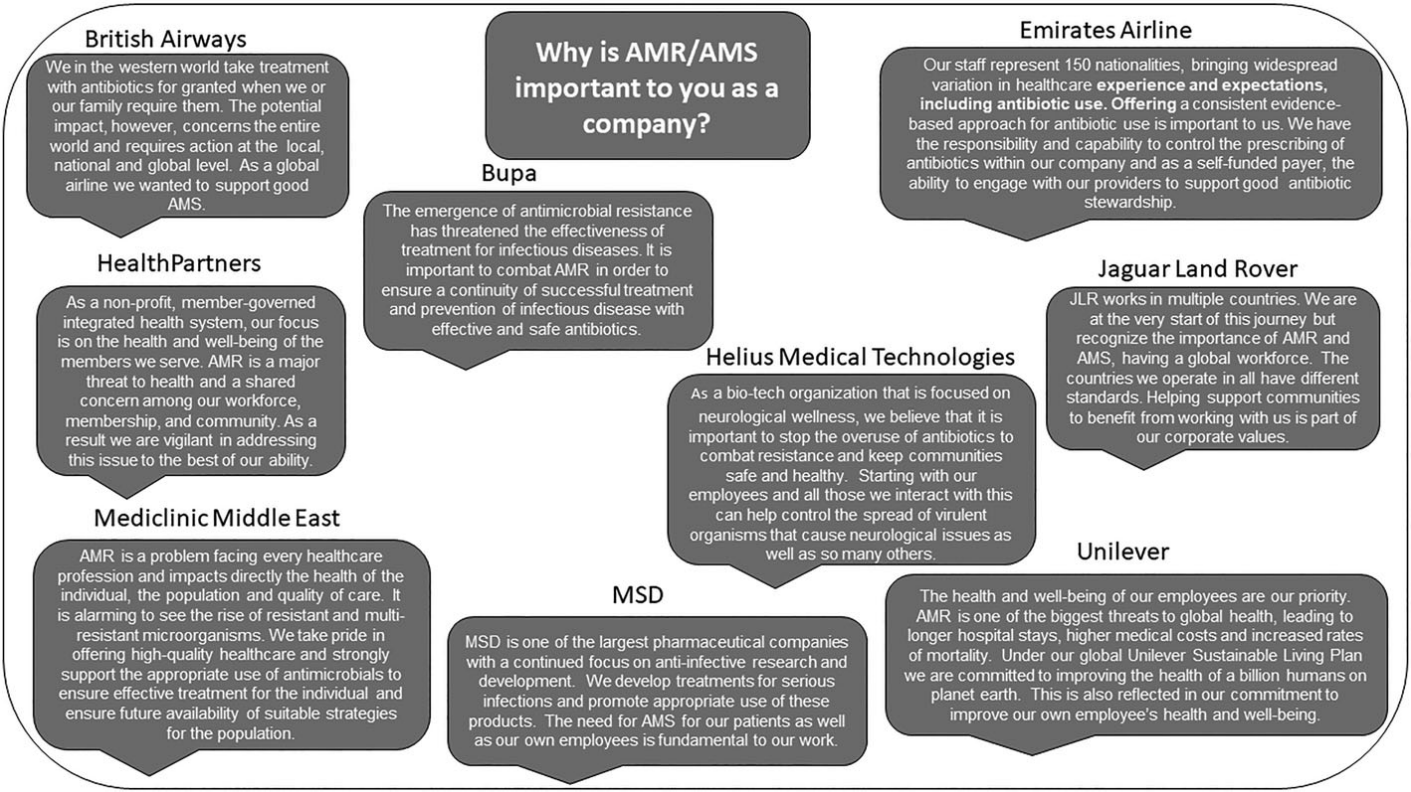
Importance of AMR and AMS. Merck & Co., Inc., Kenilworth, NJ, USA (known as MSD outside of the US and Canada). AMR, antimicrobial resistance; AMS, antimicrobial stewardship.

The Global Chief Medical Officers' Network (GCMON) is a network of corporate leaders from 58 companies that employ approximately 9 million people across the globe. The GCMON focuses on the health and well-being of their employees and the communities they serve. AMR and AMS were prioritized by the GCMON (Figure 1), and the AMR Pledge,^20^ based on the Centers for Disease Control and Prevention's Core Elements of Outpatient Antibiotic Stewardship,^17^ was developed. Four core elements of AMS are part of the Pledge: leadership commitment, action, tracking and reporting, and education. Signing the Pledge is an informal commitment demonstrating that the company is engaging in approaches and practices that support AMS. Leadership commitment has been shown to be instrumental to the success of various AMS initiatives, and signing the Pledge helps to demonstrate such commitment. However, leadership commitment goes beyond simply signing the Pledge. Adding elements of the Pledge into written company policies and assuring the commitment of senior/executive staff to not only disseminate the Pledge but also model the desired behaviors is vital to widespread adoption of the Pledge among employees. Currently, 27 GCMON companies have signed the Pledge, and other companies are invited to sign the Pledge (please contact the corresponding author).^20^

## Step 2: Perform a Gap Analysis Against Best Practices

To assess current practices, the GCMON developed a gap analysis checklist (Table 2), which was adapted for use to assess AMS activities in the employer setting from the GCMON AMR Pledge.^20^ Performing a gap analysis against best practice approaches allows companies to determine where to focus efforts related to infection prevention, awareness of AMR, and AMS. Based on the results of the gap analysis, companies can establish a baseline and identify which actions will best fit their work environment, in addition to determining where modifications of current practices may be needed. The gap analysis also allows documentation of the rationale behind a decision not to implement a particular element of the Pledge. The gap analysis should be conducted periodically to determine whether any changes are needed.

**Table 2.** Gap Analysis Checklist for Antimicrobial Stewardship Best Practices

Many companies use third-party providers to address gaps and accomplish tasks associated with the Pledge. In these cases, the company can contract (service-level agreements) with third-party providers to ensure progress is being made against the commitments in the Pledge. This includes establishing key performance indicators, reporting requirements, and periodic audits. For example, MSD outsources off-site immunizations. Included in the contract are terms that ensure all relevant data are collected for tracking purposes. These data are subsequently merged with claims data of other employees in order to obtain an overall depiction of their vaccination coverage.

## Step 3: Remedy the Gaps; Implement and/or Modify Standard Practices

Most tasks corresponding to the Pledge likely will be implemented internally. Practices will vary from company to company based on the company environment and culture and whether or not the company provides direct health care services to employees and/or customers (Tables 1 and 3). For example, Bupa owns Quality HealthCare, one of the largest providers of private health care services in Hong Kong, serving approximately 900,000 people yearly with 3 million clinic visits. Emirates Airline delivers direct health care services for 45,000 eligible staff and families through Emirates Medical Services, supported by more than 50 employed physicians, internal pharmacies, and a laboratory. HealthPartners, an integrated health system with a health plan membership of 1.8 million members, provides direct medical services to approximately 1 million members who are seen in HealthPartners' clinics. Mediclinic Middle East provides hospital and clinic services in addition to radiology and laboratory services and pharmacies. Unilever facilitates access to health care services for its employees, including at hospitals on plantations and on-site clinics at many locations. These companies can implement actions that directly impact the medical care their employees and/or customers receive. Other companies provide on-site clinics for employees who become ill or injured on the job.

**Table 3.** Global Chief Medical Officers' Network Company Antimicrobial Stewardship Practices, Metrics, and Reporting

Following guidelines for appropriate antibiotic use is important to minimize AMR. The companies providing health care services to customers routinely develop and/or follow such guidelines. AMS and/or infection control committees have been formed in their hospitals and clinics. These committees monitor antibiotic use and adherence to guidelines and provide clinical education for their care providers to ensure appropriate antibiotic use.

All the companies provide education about AMR and AMS to their employees. For example, employees of HealthPartners who are part of medical care teams are required to complete online AMR/AMS education modules. On an annual basis, 100% annual compliance has been achieved. Mediclinic Middle East holds formal continuing medical education events for its doctors and nurses. At Unilever, health care workers are educated on AMR and the importance of AMS. These individuals help to stress the importance of hand hygiene and provide education to other employees on the need for immunizations.

Leveraging existing platforms to raise awareness about AMR and AMS is beneficial because many competing priorities exist and obtaining corporate attention for this topic can be difficult. British Airways includes such education in its Health and Wellbeing program, providing travel guidance and immunizations to more than 20,000 crew members. They are advised on good AMS practices and specifically cautioned against buying antibiotics abroad. Emirates has published articles on safe and appropriate antibiotic use in their staff newspaper in addition to articles warning about the risks of taking over-the-counter antibiotics in different geographies. Helius Medical Technologies includes information on AMR/AMS in its new-hire training and its Employee Handbook. Jaguar Land Rover includes AMR/AMS on its corporate Wellbeing Agenda. MSD is launching an AMR/AMS curriculum for employees through an existing e-learning platform.

Preventing infections decreases the need for antibiotics, and adherence to recommended vaccine schedules is an essential part of this movement. To further increase immunization rates for employees, some companies modified their existing practices. Emirates now offers influenza vaccination pop-up service at flight arrivals for cabin crew and pilots, in addition to a 24-hour influenza immunization service for a 2-week period in preparation for influenza season to accommodate late-night flight crews. A significant increase in uptake was achieved in addition to positive feedback. MSD offers influenza, pneumococcal, and varicella zoster vaccinations for eligible employees at large off-site employee meetings, resulting in a 20% increase in vaccination rates among their field-based organizations.

Facilitating effective hand hygiene is another strategy to prevent infections and thus, reduce the need for antibiotics. One measure implemented by several organizations is the strategic placement of hand sanitizer to minimize the spread of infection. Likewise, posters are displayed to encourage handwashing, arm-sneezing, and minimal mouth-eye-nose touching and touching of public surfaces, as well as requesting that employees work from home when ill. With the goal of reducing requests for unnecessary antibiotics, Bupa exhibits posters of viral upper respiratory tract infections in clinics, emphasizing that they are not effectively treated with antibiotics.

Another means of enhancing AMS is through external collaborations that benefit the company and the community. Bupa conducted a retrospective study evaluating antibiotic prescriptions in private primary care settings in Hong Kong (70% of outpatient services are private). Results provided a baseline for current antibiotic use and identified areas for improvement. Emirates Medical Services initiated an endeavor aimed at sharing formularies, antibiotic resistance information, and prescribing data with select key external high-volume providers. Further, they discuss and come to agreement on best practice guidelines. The chief medical officer (CMO) of Helius Medical Technologies writes a column on health issues for pilots and dedicated an article that reached several hundred thousand aviators to the topic of AMR.^21^

Not unexpectedly, some difficulties were encountered in these endeavors. Emirates faced social expectations around the use of antibiotics because of cultural and experiential differences. Their cabin crew is highly multicultural, representing approximately 150 nationalities with different health care expectations. Some come from countries that offer antibiotics readily, frequently without requirement for a prescription. Employee feedback on their AMS program is often negative because of this high expectation to receive antibiotics even when unnecessary. Overall, Emirates' initiatives have been successful, in part because of internal communication, education and auditing, or tracking internal prescribing habits and rates. Similarly, at Unilever's hospitals on African plantations and on-site clinics, patient requests for antibiotics to treat viral infections were overcome by medical presentations at outpatient clinics every morning and one-on-one sessions with patients. In addition, clinicians demonstrated a united front to prevent going from one doctor to another to obtain the desired prescription.

## Step 4: Measure and Report Outcomes

In order to build and maintain interest in AMS, measurement and reporting of outcomes is vital. This helps to ensure efficient and effective resource utilization. Companies need to consider the audience for and frequency of reports. Reporting to external audiences is likely appropriate on a yearly basis and may be included in an annual corporate responsibility and sustainability report. However, reporting to internal audiences may need to occur more frequently, particularly if the company hopes to affect behavior change and build enthusiasm for continued initiatives. A summary of metrics and reporting for the GCMON companies is provided in Table 3.

The ability to measure and report outcomes is not limited to companies that provide health care services to their employees and/or customers. “Non-health care” companies can show meaningful impact of AMS efforts when appropriate data are captured. The most common metrics for such companies in the GCMON include vaccination rates, number of employees completing training programs, and hand hygiene compliance audits.

Companies that provide health care services directly can disseminate guidance on treatment of common infections and perform audits of prescriber adherence to the guidelines. Results of these audits are subsequently reported to the prescriber as a continuous quality improvement mechanism. For example, because Emirates Medical Services employs its own doctors and pharmacists and uses its own electronic medical records, it has the significant benefit of obtaining data from its own systems, allowing for evaluation of individual clinicians' antibiotic prescribing. At Emirates, the performance of doctors within the company's medical services is determined in part based on the volume of antibiotics prescribed and the disease/condition for which they were prescribed. Emirates is also self-insured, allowing it to evaluate its insurance data on utilization of external health care facilities.

Obtaining data for metrics pertaining to AMS can be challenging. When retrieving data on antibiotic prescriptions, Bupa finds that multiple diagnoses are sometimes recorded in a single consultation, making it difficult to identify the indication for which the antibiotic is prescribed. Also challenging is retrieving data from their very large database (because of limitations of the current information technology infrastructure). When determining which metrics to track, companies should ensure they have the means to easily obtain accurate data within their existing infrastructure.

## Lack of Awareness of AMR Is a Common Barrier

One common theme across the contributing companies was the general lack of awareness among key executives that AMR is a global priority. Including AMR/AMS as a key topic in corporate conversations is difficult. Furthermore, some companies are not in the health care space and have difficulty convincing key stakeholders of the relevance of this topic to their business. Emirates stressed information from the WHO and the United Nations in order to gain approval from their leadership. Jaguar Land Rover was able to obtain commitment from its board members by emphasizing that they should take an interest in their employees' residential communities.

Continuously engaging participation of senior executives is important to create enthusiasm around AMS and influence the workforce. The CMO of Emirates stresses the importance of having a good relationship with corporate communications to facilitate education and messaging. Helius' CMO addresses employees on AMS, disseminates information, and is available to answer questions. Through its AMS Council, composed of senior-level executives, MSD ensures that company-wide initiatives are aligned. Quarterly meetings allow for senior-level endorsement, when needed, and help to maintain momentum for AMS efforts.

## Behavioral Change and Antimicrobial Stewardship

Implementing corporate AMS initiatives is not without stumbling blocks. Limitations of the historical tools in the global fight against AMR became apparent as the “superbug crisis” sparked calls from national governments and international organizations to both find new treatments and preserve the effectiveness of existing and future antimicrobials.^4,22^ The prevailing strategies define AMR as a one-health problem (human, animal, and environmental) and involve a portfolio of supply-side and demand-side responses.^23^ Supply-side responses refer to efforts to stimulate continued research and development of novel antimicrobials, vaccines, and antimicrobial alternatives and are outside the scope of this paper. Demand-side responses deal with interventions and policies that focus on infection prevention strategies, behavior of health care providers (eg, prescribing), and treatment-seeking behavior among patients. Both provider and patient behaviors need to be considered in order to improve AMS. Indeed, patient expectations and satisfaction influence clinicians' prescribing behavior, potentially causing inappropriate prescribing.^24^ Hence, employers can play a substantial role in educating employees, influencing patient expectations for antibiotics, and supporting clinicians to make clinically-appropriate prescribing decisions in promoting AMS.

The main question an employer needs to answer is “What are the behavioral facilitators and barriers to better antibiotic prescribing?” The need for multifaceted interventions targeting multiple influences on behavior to promote responsible antibiotic use has been emphasized.^25^ Many public health experts favor traditional education and awareness campaigns. Conversely, many behavioral economists use the decision-making biases literature to design interventions that “nudge” individuals to change their behaviors.^26^ Many companies have signs above the sinks in the restrooms to encourage effective handwashing, for example.

Several traditional/reflective education and training interventions have been trialed, and some have shown significant positive outcomes for antibiotic prescribing.^27,28^ Such interventions have commonly included skills training for clinicians; for example, in enhanced communication and the use of point-of-care diagnostics. Global health action plans also address this element through education and awareness-raising activities.^1^ These interventions, however, require a significant amount of training for clinicians and may not be appealing to or feasible for those with high workloads. This should be taken into consideration when deciding how to effectively and efficiently increase awareness among employees amid many competing priorities.

A recent review of the behavioral drivers of patient antibiotic consumption proposed that interventions that require less reflective thought and more environmental cues on how to respond to symptoms of self-limiting infections could have a positive impact on behavior.^29^ Such “nudging” interventions aim to trigger automatic responses. One trial delivered a 19.7% reduction in antibiotic prescribing simply by displaying “public commitment posters” in consulting rooms.^30^ Public commitments are hypothesized to work through a desire for self-consistency (a motivation to align our behavior with previous decisions, especially those that are made public). The simplicity and success of this intervention creates potential for a low-cost, scalable intervention that warrants further testing in other settings and contexts. As mentioned earlier, several GCMON companies display posters (eg, handwashing) with messages that could be considered “nudging” interventions. Moreover, the AMR Pledge itself serves as a public commitment.

In another trial, provision of social norm feedback to high prescribers of antibiotics in general practice reduced inappropriate prescribing.^31^ In the feedback intervention group, every general practitioner was sent a letter from England's CMO stating that their practice was prescribing antibiotics at a higher rate than 80% of practices in its National Health Services Local Area Team. A similar intervention nudge successfully used a digital channel—emails to clinicians that compared their antibiotic prescribing rates with those of “top performers” (those with the lowest inappropriate prescribing rates).^32^ The prescribing feedback that Emirates provides to its clinicians as part of their performance evaluation is akin to this. Another example includes providing employee immunization rates by department.

## Conclusion

The private sector has the opportunity to support the health and well-being of its employees and communities by promoting AMS. Empowering individuals to become better antimicrobial stewards is advantageous for helping to address the significant global public health threat of AMR. Signing the AMR Pledge is an important step toward encouraging AMS because it acts as a public commitment, encouraging companies to take responsibility for honoring its directives.

An analogous pledge, the Antibiotic Guardian (AG) campaign in the United Kingdom, showed promising results.^14^ AGs are health care professionals and members of the public who may choose one of several specific pledges to use antibiotics responsibly. AGs were inspired to reduce AMR and believed that they fulfilled their pledges. Because of its success, the AG campaign has since been expanded to additional countries.^15^ One success factor of the AG campaign was thought to be its group commitment compared to an individual acting alone.^15^ The GCMON AMR Pledge facilitates a group endeavor to enhance AMS among member companies' employees.

To the authors' knowledge, this is the first manuscript describing how the private sector can support AMS using real-world examples from companies across multiple sectors. Some examples employ “nudging” behavioral techniques, such as placing hand sanitizer in convenient locations and displaying posters. Others are more traditional and involve incorporating education on AMR/AMS into existing platforms. Expanding on behavioral change techniques to complement more traditional methods can be a powerful means to reduce inappropriate antibiotic prescribing.

Although all GCMON companies were given an opportunity to participate in this paper, a limitation of this evaluation is that input was received from only 9 companies, many of which are involved in health care. A broader representation of different sectors could result in different findings and additional recommendations. Most of the companies reside in the United States, United Kingdom, and Middle East. Broader global representation also could affect the results of paper.

As AMR escalates, community education and efforts to improve antibiotic use are critical. By signing the AMR Pledge, assessing current standards, implementing best practices, and measuring and reporting outcomes, companies can partner with the broader global health care community to curb AMR and promote AMS. Both large and small companies can make a difference to advance AMS and improve the health and well-being of their employees.
